# A Prospective Assessment of the Vesical Imaging Reporting and Data System in the Diagnosis of Muscle-Invasive Bladder Cancer

**DOI:** 10.7759/cureus.83068

**Published:** 2025-04-27

**Authors:** Ahsan Ahmad, Nikhil Ranjan, Gaurav Babelay, Kumar Dheeraj

**Affiliations:** 1 Department of Urology, Indira Gandhi Institute of Medical Sciences, Patna, IND

**Keywords:** bladder cancer, mp-mri, mri, multiparametric magnetic resonance imaging, muscle invasive bladder cancer, non-muscle-invasive bladder cancer, virads

## Abstract

Background

Worldwide, bladder cancer is one of the more common types of cancer. This study was conducted to evaluate the Vesical Imaging Reporting and Data System (VI-RADS) score for the diagnosis of muscle-invasive bladder cancer (MIBC). Additionally, the VI-RADS scores were compared with various relevant parameters and cancer stages.

Materials and methods

A prospective observational study design was used. The study was carried out in the Urology Department of the Indira Gandhi Institute of Medical Sciences (IGIMS) in Patna, India. It was conducted over a period of one and a half years, i.e., from December 2022 to July 2024. Overall, 71 patients were enrolled in the study.

Results

With a cut-off score of ≥3 for T2 (muscle-invasive), sensitivity and specificity were 93.75% (79.19% to 99.23%) and 76.92% (60.67% to 88.87%), respectively. With a cut-off score of VI-RADS ≥4 for muscle invasion, sensitivity and specificity were 62.5% (43.69% to 78.90%) and 89.74% (75.78% to 97.13%), respectively. VI-RADS scores of 1 and 2 were fairly accurate for non-muscle invasion, while scores of 4 and 5 were highly predictive of muscle invasion. A VI-RADS score of 3 remained the grey zone.

Conclusion

It is easy to interpret the VI-RADS score and assess detrusor muscle invasion. The present study on VI-RADS shows reliability for local staging and for differentiating non-MIBC (NMIBC) and MIBC. Among the VI-RADS scores, it was found that the result was statistically significant between high-grade and low-grade cancer, with a p-value <0.001.

## Introduction

Worldwide, cancer of the bladder tends to be the 10th most common type of cancer. It has been noted that, as people age, the likelihood of developing a cancerous bladder increases. The male-to-female ratio of urinary bladder malignancy is 3:4 [1]. In terms of disease progression, 30% of cases of non-muscle-invasive bladder cancer (NMIBC) progress to invasive disease after initial therapy, and 80% of cases relapse within five to seven years [2,3]. Upon radical cystectomy, the five-year survival rate for T2 tumors is 68%; however, for locally advanced tumors, it is only 25%-30% [4,5].

The initial treatment for muscle-invasive bladder cancer (MIBC) was radical cystectomy, whether performed along with neoadjuvant chemotherapy or immunotherapy, or not. Computed tomography (CT) scans tend to assess the urothelium of the upper tract and also determine whether the disease has progressed locally [6,7]. Understaging may result from insufficient removal of the muscle base in transurethral resection of bladder tumors (TURBTs), which is operator-dependent. After TURBT, persistent tumors vary significantly according to experience [8,9].

Due to the strong operator dependence of the aforementioned modalities, a multimodal strategy is necessary to reduce error from a single test. Staging discrepancies between clinical and pathological conditions are frequent. Multi-parametric magnetic resonance imaging (MpMRI) aids in bridging these differences in local staging. It has been shown in many studies that dynamic contrast-enhanced imaging (DCEI) and diffusion-weighted imaging (DWI) help differentiate between superficial and muscle-invasive lesions [10,11]. MRI further depicted the involvement of lymph nodes in the metastasis of the pelvis [12,13].

The Vesical Imaging Reporting and Data System (VI-RADS) was thought to be a revolutionary technique for visualizing cancer of the bladder. It helps in better assessment of lesions of the bladder and reduces disparity among radiologists' observations by giving a more objective approach [14].

Thus, there is a need to assess VI-RADS in the population with bladder cancer to report its outcome with reliability. Therefore, this study was done to evaluate this scoring system for the diagnosis of MIBC among participants.

## Materials and methods

Study design

The study was conducted to evaluate the VI-RADS score for the diagnosis of MIBC. Additionally, the scores of VI-RADS were compared with different relevant parameters and stages of cancer. The specificity and sensitivity of the scores were also determined with different scores of VI-RADS. The research was intended to be prospective and observational. The study was carried out at the Indira Gandhi Institute of Medical Sciences (IGIMS) Urology Department in Patna, India. The study was conducted for one and a half years, i.e., from December 2022 to July 2024.

Study population

This study included a total of 71 patients. Patients who had a suspected bladder mass identified clinically using urine cytology tests and cystoscopy, or by any radiological test such as an ultrasound, CT scan, or MRI, and who were willing to give informed consent, were eligible to participate. The exclusion criteria also included patients with non-transitional cell carcinoma, variant histology, participants who were allowed to participate due to the presence of some co-morbidities, patients who had previously undergone surgery, chemotherapy, or radiotherapy for bladder tumors, and patients who were not MRI-compatible.

Data collection

The study data include the gender of participants, the grade of cancer among participants, and the stages of tumor among participants. The data were further segregated according to the scores of VI-RADS obtained among participants.

Study procedure

Following appropriate bladder distension, all eligible patients underwent an mpMRI. Every patient received the identical 3 Tesla scan mpMRI protocol (Discovery 750; GE Healthcare, Milan, Italy). T2-weighted sequences were obtained in the axial, coronal, and sagittal planes, as per VI-RADS; DWIs with high b values (b = 0-800-1000, up to 2000 s/mm²) were obtained in the axial plane, and DCEIs were obtained in the axial plane with a temporal resolution of five seconds. To achieve sufficient bladder distension, patients were given an injectable antispasmodic medication and told to consume 500-1000 mL of water 30 minutes before the test. Two urogenital radiologists, who were blind to the clinical history, examined every examination. Each lesion was given a VI-RADS score (1-5) by both readers, with a maximum of three points per patient. Only the patient with the highest VI-RADS score was taken into consideration. It was believed that MIBC would be defined by a VI-RADS cutoff score of ≥3. Assuming the view of the most seasoned reader to be the final one, disagreements were settled by consensus.

In the presence of several bladder lesions, the highest VI-RADS scores were recorded. Muscle invasion was predicted by VI-RADS values of ≥3 and ≥4. TURBT was performed, followed by cystoscopy with a bipolar resectoscope under general anesthesia. Histopathological examination (HPE) was performed on all deep biopsies and resected tumor specimens. The pre-operative VI-RADS scores and the final HPEs of each patient were compared and examined. Re-TURBT was performed within six weeks in accordance with the European Association of Urology (EAU) guidelines. For all T1 patients and those with HG-Ta (high-grade Ta) and missing muscularis propria in the resection specimen, re-TURBT was carried out at the scar site or sites of the first resection within two to six weeks following the initial procedure.

Statistical analysis

The following simple formula would be used for calculating the adequate sample size in a prevalence study [15]: $n = \frac{Z^2 P (1 - P)}{d^2}$, where n is the sample size, Z is the statistic corresponding to the level of confidence, P is the expected prevalence, and d is precision.

In this study, an analysis of statistics was done using IBM SPSS Statistics for Windows, Version 24 (Released 2016; IBM Corp., Armonk, NY, USA). The receiver operating characteristic (ROC) curve is used to compute the area under the curve (AUC). Detrusor muscle invasion was predicted using cutoff VI-RADS scores of 3 and 4. Data were presented as either n (%), or n (interval ratio), or % (95% confidence interval). The Chi-square statistic has been employed to obtain the p-value. The significance of the analysis was defined with a p-value of less than 0.05.

Ethical clearance

The Institutional Ethics Committee (IEC) of IGIMS, Patna, India, has approved the ethics under letter number 847/IEC/IGIMS/2022, dated December 10, 2022.

## Results

Most of the participating patients were male, as compared to females. In VI-RADS score 4, 15 (93.75%) of patients had high-grade cancer, while only one (6.25%) patient had low-grade cancer. In the VI-RADS score 5, all eight (100%) patients had high-grade cancer, and no patients had low-grade cancer. Among the VI-RADS scoring, it was found that the result was statistically significant between high-grade and low-grade cancer, with a p-value <0.001. Table 1 depicts the main characteristics of participants distributed according to VI-RADS.

**Table 1:** Distribution of characteristics according to VI-RADS score Data are presented as n (%). The Chi-square test was used to obtain the p-value, and the p-value was considered significant at less than 0.05. VI-RADS, Vesical Imaging Reporting and Data System

Characteristics	VI-RADS 1 (n = 11)	VI-RADS 2 (n = 21)	VI-RADS 3 (n = 15)	VI-RADS 4 (n = 16)	VI-RADS 5 (n = 8)	Chi-square statistic	p-value
Female	02 (18.18%)	05 (23.80%)	02 (13.33%)	05 (31.25%)	01 (12.5%)	2.03	0.72
Male	09 (81.81%)	16 (76.19%)	13 (86.66%)	11 (68.75%)	07 (87.5%)		
High-grade cancer	01 (9.09%)	11 (52.38%)	11 (73.33%)	15 (93.75%)	08 (100%)	24.06	<0.001
Low-grade cancer	10 (90.90%)	10 (47.61%)	04 (26.66%)	01 (6.25%)	00 (0%)		

**Figure 1:**
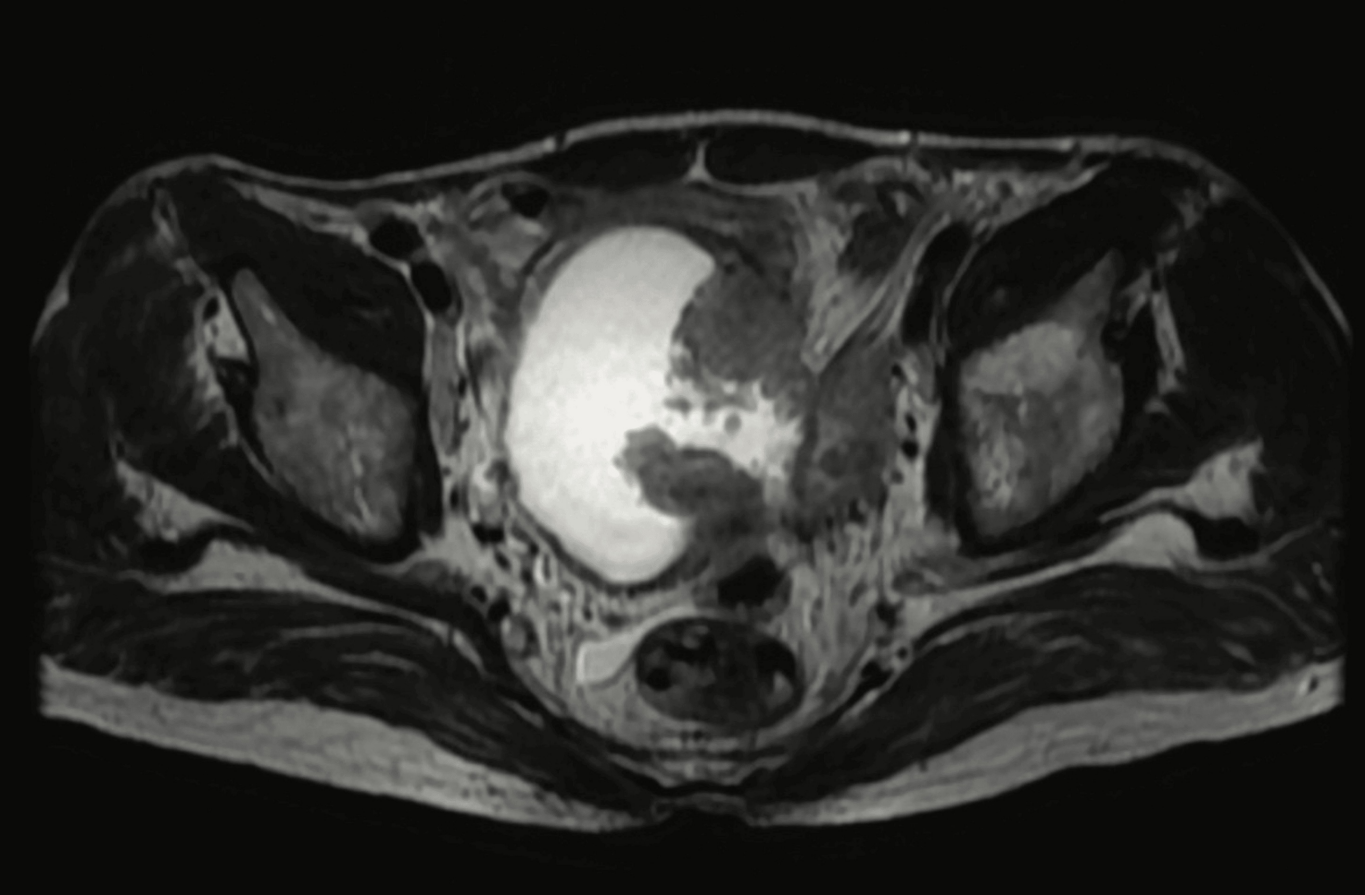
T2WI reveals that the intermediate signal tumor had invaded the whole bladder wall and spread to the extravesical fat T2WI, T2-Weighted Imaging

**Figure 2:**
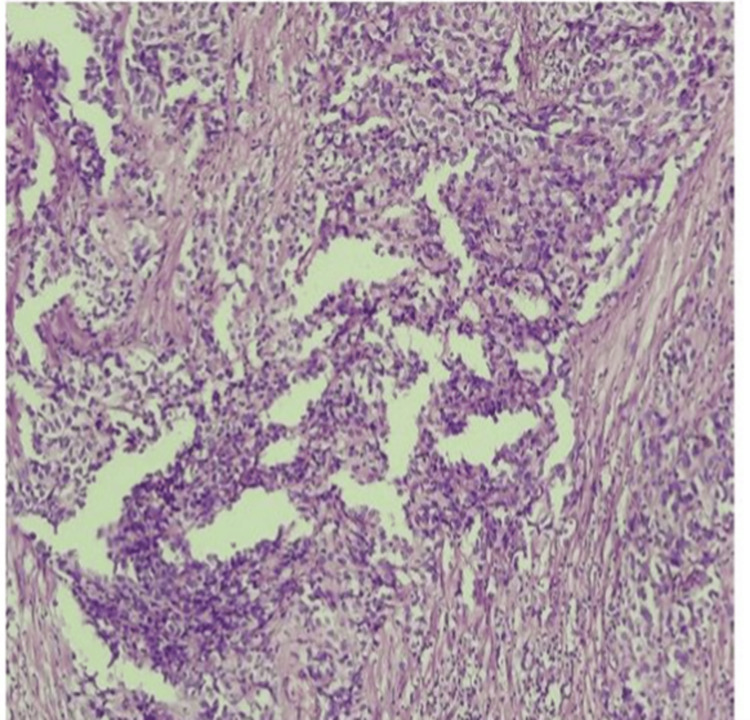
HPE (100x) shows tumour cells with muscularis propria invasion HPE, Histopathological Examination

**Figure 3:**
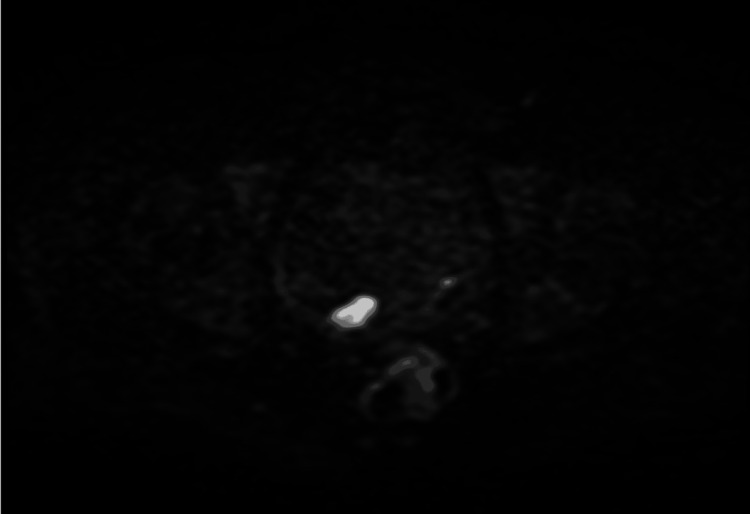
DWI (b = 1000 s/mm²) shows a tumor with hyperintense signal intensity DWI, Diffusion-Weighted Imaging

**Figure 4:**
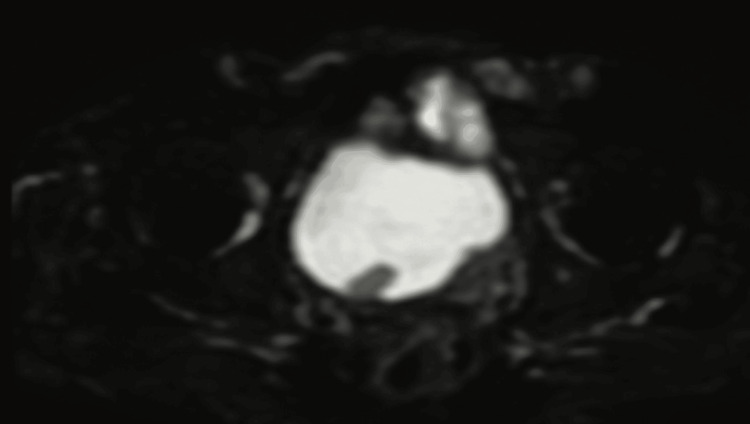
ADC map shows the same lesion as hypointense signal ADC, Apparent Diffusion Coefficient

**Figure 5:**
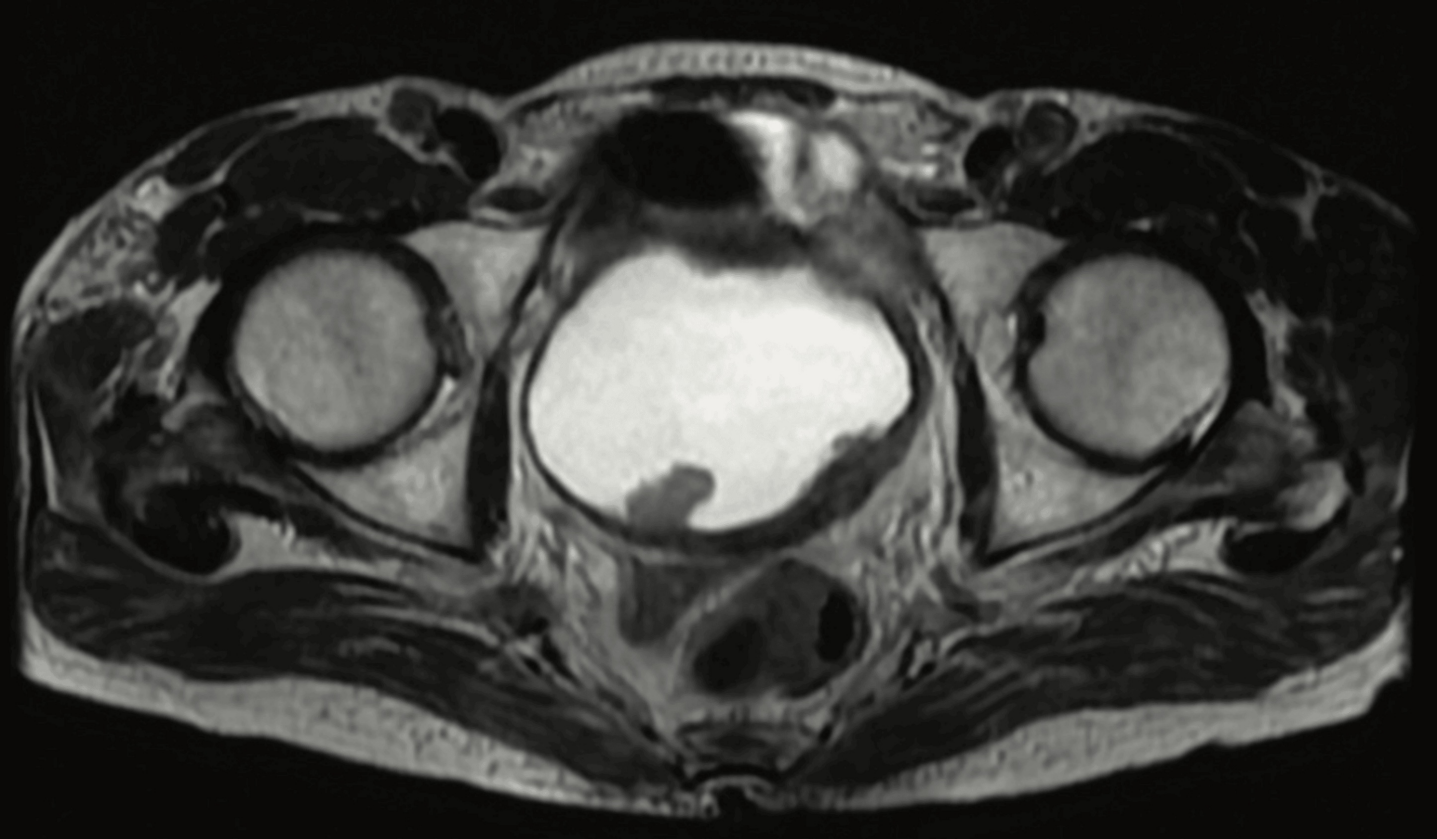
A tumor with moderate signal intensity, originating from the posterior bladder wall, is visible on T2WI (axial) T2WI, T2-Weighted Imaging

**Figure 6:**
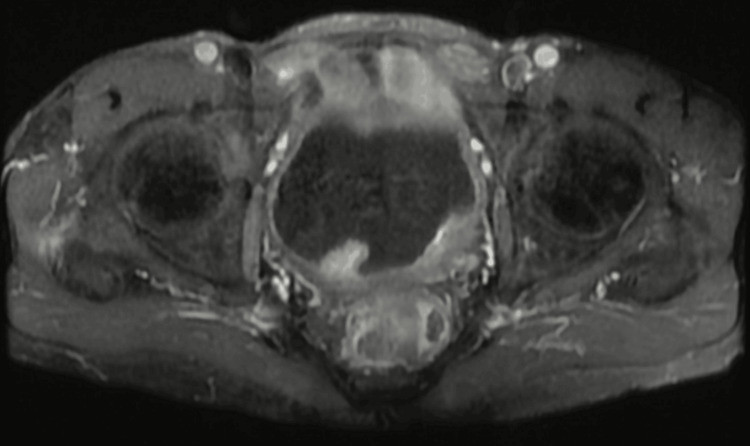
An image of DCEI DCEI, Dynamic Contrast-Enhancement Imaging

**Figure 7:**
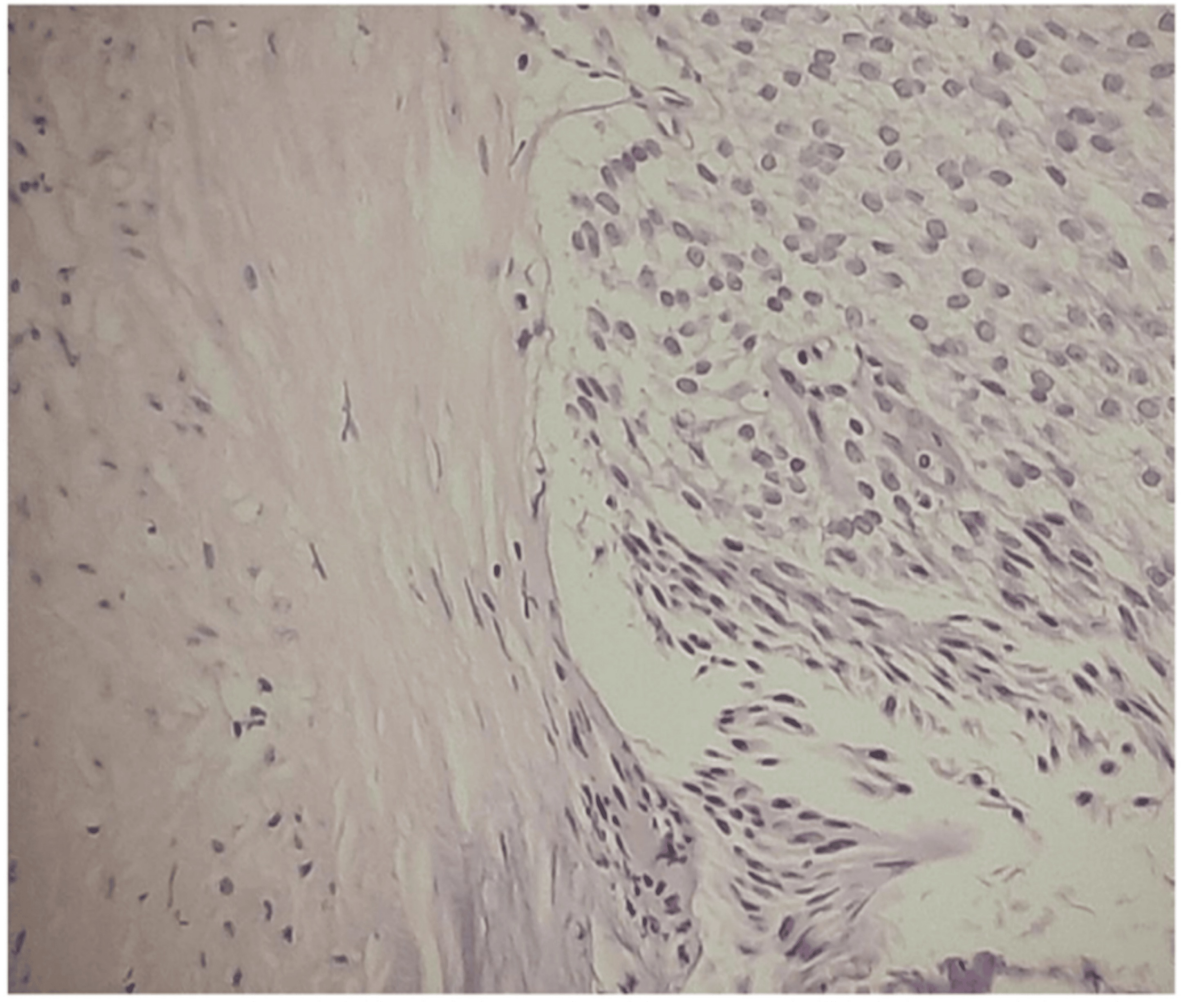
HPE (100x), post-TURBT, shows low-grade transitional cell carcinoma not involving the muscularis propria HPE, Histopathological Examination; TURBT, Transurethral Resection of Bladder Tumor

**Figure 8:**
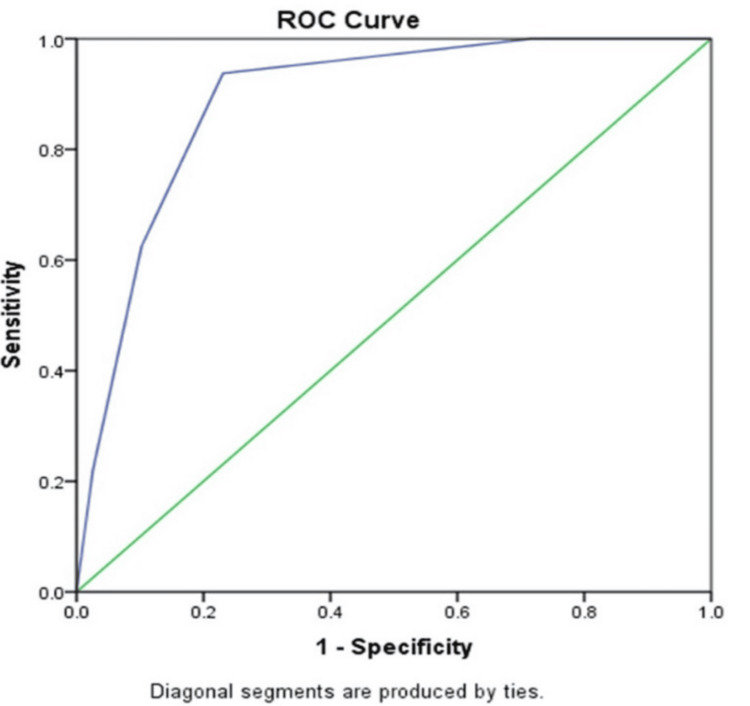
Diagrammatic representation of the ROC curve shows specificity and sensitivity, and displays an AUC value of 0.88 with a 95% CI of 0.81-0.96 The green line represents the 'reference line' drawn at 45 degrees; the blue line corresponds to the ROC curve of the diagnostic test (MpMRI with VI-RADS) ROC, Receiver Operating Characteristic; AUC, Area Under the Curve; MpMRI, Multi-parametric Magnetic Resonance Imaging; VI-RADS, Vesical Imaging Reporting and Data System

Figure 1 entails T2-weighted imaging (T2WI), revealing that the intermediate signal tumor had invaded the whole bladder wall and spread to the extravesical fat. Figure 2 (HPE, 100x) shows tumor cells with muscularis propria invasion. Figure 3 depicts DWI (b = 1000 s/mm²), showing a tumor with hyperintense signal intensity (restricted diffusion). Figure 4 represents the apparent diffusion coefficient (ADC) map, showing the same lesion as a hypointense signal. Figure 5 shows a tumor with moderate signal intensity, originating from the posterior wall of the bladder, visible on T2WI (axial). Figure 6 shows an image of DCEI. Figure 7 shows that, following TURBT, HPE (100x) reveals low-grade transitional cell carcinoma that does not involve the muscularis propria. Lastly, Figure 8 depicts a diagrammatic representation of the ROC curve, showing specificity and sensitivity, with an AUC value of 0.88 and a 95% CI of 0.81-0.96.

Table 2 represents the calculation of various parameters at cut-off values greater than or equal to 3 and 4. When the cut-off value of VI-RADS was taken as 3 for the diagnosis of MIBC, the sensitivity of mpMRI was 93.75% (CI: 79.19%-99.23%), specificity was 76.92% (CI: 60.67%-88.87%), the positive likelihood ratio was 4.06 (CI: 2.27-7.27), and the negative likelihood ratio was 0.08 (CI: 0.02-0.31). Likewise, the positive predictive value was 76% (CI: 65.11%-85.62%), the negative predictive value was 93.75% (CI: 79.50%-98.31%), and the accuracy was 84.51% (CI: 73.97%-92.00%).

**Table 2:** Calculation of various parameters at the cut-off of ≥3 and ≥4 Data are presented as either n (interval ratio) or % (95% confidence interval). VI-RADS, Vesical Imaging Reporting and Data System

Parameters	VI-RADS ≥3	VI-RADS ≥4
Sensitivity	93.75% (79.19%-99.23%)	62.5% (43.69%-78.90%)
Specificity	76.92% (60.67%-88.87%)	89.74% (75.78%-97.13%)
Positive likelihood ratio	4.06 (2.27-7.26)	6.09 (2.32-16.02)
Negative likelihood ratio	0.08 (0.02-0.31)	0.42 (0.26-0.66)
Positive predictive value	76.9% (65.11%-85.62%)	83.3% (65.54%-92.93%)
Negative predictive value	93.7% (79.50%-98.31%)	74.4% (64.81%-82.20%)
Accuracy	84.5% (73.97%-92.00%)	77.4% (66%-86.54%)

Seven (87.5%) out of eight cases with VI-RADS 5 were T2, and only one (12.5%) case was T1. Similarly, 13 (81.25%) out of 16 cases with VI-RADS 4 were reported as T2, and three (18.75%) cases were T1. Among the 15 cases with VI-RADS 3, 10 (66.67%) were T2, and five (33.33%) were T1. Out of 21 cases of VI-RADS 2, 19 (90.47%) were T1, and the remaining two (9.52%) were reported as T2. Among the 11 cases of VI-RADS 1, four (36.36%) were T1, and the remaining seven (63.64%) were Ta. Table 3 shows the staging of the tumor among VI-RADS scores.

**Table 3:** Tumor stage distribution among VI-RADS Data are presented as n (%). VI-RADS, Vesical Imaging Reporting and Data System

Stages of tumor	VI-RADS 1 (n = 11)	VI-RADS 2 (n = 21)	VI-RADS 3 (n = 15)	VI-RADS 4 (n = 16)	VI-RADS 5 (n = 8)
T1	4 (36.36%)	19 (90.47%)	5 (33.33%)	3 (18.75%)	1 (12.5%)
T2	0 (0%)	2 (9.52%)	10 (66.66%)	13 (81.25%)	7 (87.55)
Ta	7 (63.63%)	0 (0%)	0 (0%)	0 (0%)	0 (0%)

## Discussion

Bladder cancer is becoming more and more common worldwide, making it prevalent among genitourinary cancers. The mainstay of urinary bladder cancer therapy is based on the presence or absence of muscle invasion. Hence, accurate pre-operative tumor staging becomes very important for both management and prognosis. At present, CT is mostly used as an imaging modality for pre-operative staging, but it carries added radiation risk. TURBT is the gold standard; however, its accuracy is affected by multiple factors, including the absence of muscle in the specimen, the presence of multiple tumors, complications during surgery, and the skill of the physician.

Furthermore, many discrepancies and disagreements are observed between CT imaging and TURBT. It is evident from the above discussion that there is a need for a diagnostic imaging modality capable of accurately predicting the presence or absence of muscle invasion. In our study, VI-RADS scores from 1 to 5 demonstrated good accuracy in identifying muscle invasion and showed fair agreement with the pathology report. A K-value of 0.634 (95% CI: 0.44 to 0.824) suggests good diagnostic agreement between VI-RADS and pathological findings. It was also found that the ratio of muscle invasion to non-muscle invasion was only 2:1 in three VI-RADS patients, making it challenging to accurately forecast the depth of invasion.

In Del Giudice et al.'s prospective study, using a VI-RADS cut-off of ≥3, the AUC, sensitivity, and specificity were 0.94, 91.9%, and 91.1%, respectively. They had a larger sample size of 231 patients. Specificity and AUC were better than in our study; however, sensitivity was comparable [16].

Marchioni et al., with a smaller sample size and using a VI-RADS cut-off score of ≥4, showed similar sensitivity and specificity. The study had better specificity but poorer sensitivity [17].

According to Ueno et al.'s retrospective analysis, the AUC, sensitivity, and specificity were 88%, 77%, and 0.9, respectively, with a VI-RADS ≥3 threshold. Despite having a comparable sample size of 74 participants, their study demonstrated superior sensitivity and specificity compared to ours [18].

A retrospective investigation by Wang et al. demonstrated that the specificity, AUC, and sensitivity were 87.1%, 0.94, and 96.5%, respectively, using VI-RADS ≥3 as the cut-off for muscle invasion. Our study showed better sensitivity but poorer specificity and AUC [19].

So, VI-RADS, after validation, could bring a paradigm shift in bladder cancer management. Complete TURBT and early intravesical therapy should be the goal for patients who have a lower risk of muscle invasion, in accordance with a VI-RADS score of 1 or 2. This would help reduce delays in subsequent management, i.e., intravesical therapy.

The only goal in cases with VI-RADS 4 or 5, which indicate a high risk of muscle invasion, should be a biopsy of the tumor with sufficient depth, followed by early neoadjuvant chemotherapy and radical cystectomy. This will prevent the need for re-TURBT and save overall costs on the final treatment.

Limitations

The limitations of the study included that it was a single-center study with a small sample size. Henceforth, a multicentric study with a larger sample size will be required to validate the results. Also, the availability of mpMRI, its cost, and the learning curve in VI-RADS scoring and reporting are other limitations.

## Conclusions

It is easy to interpret the VI-RADS score and assess detrusor muscle invasion. The present study on VI-RADS shows reliability for local staging and differentiating NMIBC and MIBC. Among the VI-RADS scoring, it was found that the result was statistically significant between high-grade and low-grade cancer, with a p-value <0.001. In addition, the present study also highlights the potential implications of mpMRI and the VI-RADS score to redefine the role of restaging TURBT. However, a multicentric study with a larger population is required for further validation.
